# Examining lag effects between industrial land development and regional economic changes: The Netherlands experience

**DOI:** 10.1371/journal.pone.0183285

**Published:** 2017-09-06

**Authors:** Eda Ustaoglu, Carlo Lavalle

**Affiliations:** Institute for Environment and Sustainability, European Commission-Joint Research Centre, Sustainability Assessment Unit, Ispra, Italy; University of Rijeka, CROATIA

## Abstract

In most empirical applications, forecasting models for the analysis of industrial land focus on the relationship between current values of economic parameters and industrial land use. This paper aims to test this assumption by focusing on the dynamic relationship between current and lagged values of the ‘economic fundamentals’ and industrial land development. Not much effort has yet been attributed to develop land forecasting models to predict the demand for industrial land except those applying static regressions or other statistical measures. In this research, we estimated a dynamic panel data model across 40 regions from 2000 to 2008 for the Netherlands to uncover the relationship between current and lagged values of economic parameters and industrial land development. Land-use regulations such as land zoning policies, and other land-use restrictions like natural protection areas, geographical limitations in the form of water bodies or sludge areas are expected to affect supply of land, which will in turn be reflected in industrial land market outcomes. Our results suggest that gross domestic product (GDP), industrial employment, gross value added (GVA), property price, and other parameters representing demand and supply conditions in the industrial market explain industrial land developments with high significance levels. It is also shown that contrary to the current values, lagged values of the economic parameters have more sound relationships with the industrial developments in the Netherlands. The findings suggest use of lags between selected economic parameters and industrial land use in land forecasting applications.

## Introduction

Industrial land constitutes a significant part of the land value in urban areas and regions. Considering that land use reflects decisions of the private and public agents, the patterns of land development observed in an urban environment reflect the decision-making processes of those who manage scarce land resources. Industrial and commercial development policies such as the availability of subsidies and tax benefits applied to new development schemes, socio-economic growth and technological development, external trade policies, fixing of constraints or setting new allowances on new land developments have caused significant impacts on the urban land, in particular industrial land developments [1–4]. Because of the impact of such policies on spatial configuration of land use, it is imperative to understand how industrial land might evolve in an urban environment.

The dynamism of land-use change related to industrial and commercial activities is of significance to land-use models which simulate the competition between various land uses to assess possible future land developments [5,6]. Land-use modelling is central to the planning and policy making processes as it provides an understanding of how future land-use configurations might evolve under different socio-economic conditions and possible future policy alternatives [7]. Reviews of spatial land-use models can be found in Briassoulis [8] and Verburg et al. [9].

By contrast to spatial land-use models, the literature points to non-spatial models aiming at estimating land-use change as regional or country total aggregates without providing spatial location of the estimated land-use changes. These models are based on different statistical and modelling techniques including econometric regressions and other statistical measures (see Batista e Silva et al. [10] for a review), system dynamics [11] and agent-based modelling [12]. Particularly, the techniques of econometric regressions [13; 14] and other statistical measures including estimations of socio-economic indicators [15], real property value forecasts [16], trend extrapolation and density measures [10, 13] are often used in many countries to make forecasts for the demand of industrial and commercial sites. This is crucial for the countries where there is high economic growth and development with a limited supply of land resources [17, 18]. These techniques are preferred over more sophisticated alternatives considering that they are mostly based on socio-economic parameters (such as population, employment, real property price index, economic output or income) that are available for long time periods in many countries.

In some applications, the non-spatial modelling techniques are used for the estimation of future land demand to be fed into spatial land-use models. In such approaches, land-use demand projections are computed externally at the regional or country scale, and they are fed into the fine-scale land-use models. The spatial land-use models can allocate the future demand for various land uses through spatial land allocation modules [19, 20]. The models such as CLUE-S and EU-CLUE Scanner are examples of such a structured approach where externally projected land-use demand can be integrated to the spatial land allocation module [21, 22]. Land Use Scanner is another example of a spatial land allocation model that simulates future land use through integration and allocation of future demand for land provided by external sources such as sector specific models or policy constraints [23]. Regarding the application of Land Use Scanner model for the Netherlands, an internal modelling framework is applied to compute demographic and regional employment figures, which are subsequently utilised to compute net residential and industrial areas at the regional level [24]. Land Use Scanner uses these regional demands in combination with local suitability approach for each modelled land-use type to simulate future land use.

The aim of this study is to contribute to this literature by focusing on the dynamics of industrial land development estimated through the use of commonly applied economic parameters, particularly manufacturing output, economic income, industrial property price and employment. Considering that industrial property investment and subsequent demand for industrial land development are driven by market mechanism through the interaction of supply and demand, these are highly sensitive to any variations in economic conditions. The common assumption regarding industrial property markets is the effective response of the property market to land development pressures following economic growth in a region or country. However, there are a number of factors that make property market less efficient than other markets. These are imperfect market information, cyclical nature of property developments resulting in oversupply or undersupply in the market, high transaction costs, a number of development constraints, irreversibility, and illiquidity [25–27]. Considering existence of such failures, there are lags between the decision to begin real property development and its completion [27, 28].

From government’s perspective, it is important to get the information on lags between the time when an irreversible decision is undertaken and when the development’s first revenues are received. These lags may be quite long [29]. For instance, in the Netherlands, there is a gap of six to eight years between development of a land-use plan and provision of the industrial sites for development [30]. Following the development of an industrial site, the land use has almost a permanent character considering that urban re-development is very time consuming and expensive [30]. Therefore, it is important that any substantial industrial capital investment programme should be supported by various considerations such as societal needs, budgetary constraints and potential impacts of development lags on social welfare. The underdeveloped land has negative social impacts e.g. potential lack of business estates for investors, vacant lands, and soaring land and industrial property prices while overdeveloped land may result in vacancies in industrial property, overconsumption of land, and declining property prices. Without an estimation of the relationship between development lags and industrial property development, any capital investment policy could result in a non-reversible and unsustainable consequence in the long run.

From policy makers’ and planners’ perspective, it is necessary to consider how sensitive the industrial property market to economic fluctuations and development lags. However, this is a difficult task without developing a reliable forecast of industrial land demand. Therefore, it is essential to focus on statistical and econometric techniques to explain the interaction between industrial development and real estate development time lags. In this study, a dynamic panel regression approach is adopted to predict industrial land demand. The model can enable policymakers and planners to produce more effective policies and regulations to guide the real property market, tackle existing and potential problems and promote social welfare. The model can also facilitate construction stakeholders to forecast the industrial land through incorporating development lags into the model in the short to medium term to enable the industrial construction market to plan forward [31]. This would enable the construction industry and other construction stakeholders to optimise their returns from industrial investment and sustain economic development.

The research on forecasting demand for industrial or commercial land is scarce as the literature is mainly dominated by studies that focus on the drivers of urban land-use change [32, 33] and the factors governing variations in industrial property rents (or prices) [34, 35] or property developments [36]. In the scarce literature, there are studies of forecasting work for the industrial property market utilising supply and demand dynamics and prices (or rents) [37, 38]. Some examples of research focusing on the dynamics of industrial demand in relation to economic and sectorial variables are Beckers and Schuur [18], Ramirez [39] and Sing [40]. The research by Sing [40] found significant coefficients for the first three lags of the GDP variable on industrial space demand in Singapore. By contrast, Beckers and Schuur [18] focused only on the relationship between employment and industrial land demand in the Netherlands. Though the study found significant non-linear relationship between sectorial employment and land use, it does not provide any evidence of time lag effects of the employment variables on industrial land use.

The studies by Hoymann [13], and Reginster and Rounsevell [41] utilised regression models to estimate demand for urban area. The former study compared results of the residential demand predictions with the observed data through application of different statistical approaches. The latter research applied regression techniques for the prediction of urban demand, which was fed into their studied land-use model to simulate future land-use changes. Despite estimations of significant coefficients for the selected predictors of urban land use, none of their regressions considered development lags concerning the urban land demand. Among few studies of forecasting work of the industrial land, Batista e Silva et al. [10] estimated demand for industrial and commercial land for the EU countries by using a number of statistical approaches and compared prediction results with the observed data following the research by Hoymann [13]. However, the study did not consider lag effects of the economic development on the growth of industrial and commercial land use in their studied countries.

Except few studies, time lag effects of economic changes on land use have rarely been empirically assessed particularly focusing on industrial land developments. In this respect, our study attempts to contribute to the existing research through providing a dynamic analysis example to uncover the relationship between lagged values of selected economic parameters and industrial land development across 40 regions spanning 8 years (2000–2008) in the Netherlands. We use a dynamic panel data approach which enables us to integrate the spatial characteristics of the regions into the time-series analysis, and can improve the robustness of the estimates.

The remainder of the paper is organised as follows: Section 2 reviews the literature on real property development lags and construction demand forecasting methods. The data and methodology are described in section 3, and estimation results are presented in section 4. Section 5 compares the actual and the model estimation results of industrial land developments. Section 6 concludes the paper.

## Literature review

### Real property development lags

The overbuilding (and underbuilding) of real property development is well documented in the literature, particularly for commercial and residential property markets. Wheaton [42] provides evidence on office property over-buildings in the past decades in US (see also King and McCue [43]; Grebler and Burns [44]; Hendershott [45]). Barras and Ferguson [46] showed that new commercial and industrial property demand coincides with the business cycle, however there is a lag of three to four quarters between new orders and construction completions. Barras [47] examined the variables affecting demand for office space such as output prices, user cost of capital and profitability. His results suggested that lagged output is the most important variable. He also stated that the long production period between new building orders and completions are the crucial endogenous factors causing building cycles. DiPasquale and Wheaton [25] have demonstrated that stock adjustment through new construction is much slower than price adjustment in real property markets when there is an external shock in the economy. In the long run, the stock adjusts gradually considering the lags in the delivery of new stock. Capital investment decisions are based on the forecasts of property prices at the time of new construction completions [42]. This implies that any external shock to the economy is first reflected in the real property prices and rents but not in physical assets.

Grenadier [48] (cited in Ball et al. [49]: 205) argues that ‘the lag explanations assume myopic behaviour of developers and therefore they should take into account the future state of the market’. Ganesan and Tse [50] examined the lead-lag relationship between construction activity and the aggregate economy in Hong Kong. The study concluded that economic output results in construction flow. However, the initial impact of a change in GDP would be on the change in real estate investment projects but not on the level of construction output considering that construction activity is very sensitive to credit conditions and land development restrictions. Wheaton [42] examined the future expectations of agents in a stock-flow model where development lags, the degree of durability and market elasticities can vary. He concluded that stock-flow models with myopic behaviour present high sensitivities to all of these parameters and that rational model can also generate cyclical behaviour based on exogenous structure on the market’s operation that leads to historical dependence.

The existence of real property construction lags was examined by Spiegel [51] in the framework of a general equilibrium model for the residential housing market to explain the housing construction cycles in relation to expected housing returns. Barras [52] alternatively constructed a dynamic model of building cycles for the London office market. The adjustment process in his model is determined by three types of lags: the occupiers’ response to change in rents, the developers’ response to changes in demand through rent adjustment process, and the construction delay between starts and completions (see also Spiegel [51]). The existence of lags in the demand and supply relationship of the office market in Stockholm is considered through application of error correction models in the study of Englund et al. [53]. The lagged values of rents, vacancy rates and employment variables were found to be significant in their three-equation system model. A similar study by Hendershott et al. [37] analysed long run and short-run rent, vacancy and supply adjustments in the US retail market. In the model estimations, various lags were considered for the variables utilised in systems of demand and supply equations. Some other recent examples on the existence of lagged variables utilised in explaining the adjustment processes in demand and supply relationship in commercial and industrial property markets can be found in Fuerst and Grandy [54], White and Ke [55], Mesthrige [56], Bruneau and Cherfauh [57].

### Construction demand forecasting methods

Various statistical models have been established and tested for forecasting the demand for construction activities. There are two types of models, namely, the univariate and the causal models [58]. The univariate models are based on the past values of time series of the selected variable. The common univariate models applied in a wide range of studies to forecast construction demand are Box-Jenkins [16] and exponential smoothing techniques (see Jiang and Liu [59] for detailed review). The causal modelling techniques, by contrast, consider a number of related variables to explain the construction demand while forecasting the future construction activity. The causal models cover the classical multiple regression and advance multivariate regression techniques. Multiple regression models were applied for various scenarios to forecast sectorial construction demand in Thailand [60] to investigate the relationship between space demand and residential property investment [61] and private housing demand [62] in Hong Kong.

The advanced multivariate models to predict construction demand are vector autoregressive and vector error correction models, which can estimate a selected regressand based on its own lags and the lags of other variables. There are few studies in the literature applying these techniques for the construction demand forecasting as these techniques are mainly utilised for the estimation of various economic indicators including unemployment, interest rate, exchange rate, and housing prices (see Ng et al. [63] for a review). There are recent studies applying vector error correction modelling techniques to forecast construction demand including private construction investment [63, 64] and labour demand [65] in the Hong Kong construction market, and changes in construction demand following the economic crises in Australia [59].

Despite the fact that vector error correction models captures both the short-term dynamics as well as the long-term equilibrium adjustment process among the variables, these models cannot explain spatial and market-based differences across the regions. These techniques assume general equilibrium throughout the entire land market, implying homogeneity of the estimated coefficients across different submarkets and different regions. The existence of submarkets and locational differences of the regions can indicate structural variations of demand for industrial land considering that each submarket has specific demand and supply conditions, as well as different locational characteristics. The consideration of these factors is complex in time-series analysis and further improvements are necessary for the estimation of dynamic models. In our model, we have utilised a dynamic panel data approach by including a number of location and area specific characteristics as potential instruments to consider spatial heterogeneity in the study area. In this, our model provides a significant improvement over time-series models (i.e. univariate and multivariate models) which do not consider such regional and spatial variations.

## Data and methodology

### Methodology

#### Modelling industrial land market dynamics

The growth of economic activities (i.e. economic output and employment) in cities and regions results in physical changes in industrial land in the medium to long-term following an increase in demand for new industrial property developments. A considerable correlation between industrial and residential property and the economy is confirmed by the study of Wheaton [42]. A similar study by Ball and Wood [66] found evidence between aggregate economy and construction activity in the real property sector (see also Barras [52]). Therefore, the demand for industrial space can be represented as a function of economic activity in urban areas and regions. The increase in demand for new industrial space increases rental prices of the industrial estates in the short-run. Ball et al. [49] argued that changes in the nature of production and output generally results in more or less intensive use of space for a given level of output. Therefore, in the short-run firms do not change their demand for physical space but they adjust the existing space according to their new production needs.

In the economic literature, the effect of transport infrastructure on industrial land development is incorporated as an additional input in the production function since accessibility to certain activities raises productivity or reduces cost of production [67, 68]. Therefore, accessibility can also be considered as an additional factor determining the demand for industrial land through influencing the profitability of industrial production. Given this literature, we can suggest that changes in economic activities, accessibility and rental prices of real property influence demand for industrial space in the market where the demand function can be stated as:

$$D_{i,t}^{L}=f\left(E_{i,t},R_{i,t},A_{i,t}\right)$$ (1)

where E_i,t_ represents economic parameters (i.e. GDP, industrial GVA and industrial employment), A_i,t_ is the accessibility indicator which will be elaborated in future sections, and R_i,t_ is the industrial rental price of the i th region at time t. If there is excess demand for the existing industrial estates, it will rise the prices and rental prices of the industrial properties. The increase in rents and price for industrial space provides an incentive for developers to initiate new investments in the market. Therefore, developers will generate new construction investments in the long run. Property developers generally make investment decisions based on the prices in the recent past period [69].

Following Wheaton [42], we can state that supply depends on historical prices, P_i,t-s,_ and asset prices at the time of the delivery, P_i,t_, as industrial properties are occupied by multiple tenants. The accessibility to industrial activities (i.e. jobs) is an important determinant of industrial land price, which consequently influences supply of land considering that land developers take into account the issues of accessibility of land uses and their price for their investment decisions in the market. A zone which has higher accessibility to industrial jobs imply that the subject zone has higher industrial land prices compared to others having lower accessibility values. Therefore, an accessibility measure, A_i,t_ can be included in the supply function as an independent variable.

In order to obtain an understanding of the impact of industrial land conversions to other uses (e.g. social facilities, residential uses) on the development of industrial land use, we include a variable, C_i,t_, in the supply equation indicating the areas of industrial land existing at time (t-s) and converted to other uses at time t. Considering that land supply is also dependent on the developed space in the previous periods (t-s), land-use restrictions such as land zoning policies imposed by local or national authorities, Z_i,t_, and natural limitations such as protected areas, water bodies or sludge areas, N_i,t_, land supply function can be provided as:

$$S_{i,t}^{L}=f\left(P_{i,t},P_{i,t-s},R_{i,t},R_{i,t-s},A_{i,t},C_{i,t},Z_{i,t},N_{i,t},S_{i,t-s}\right)$$ (2)

It can be suggested that industrial property supply is also determined by other factors such as construction costs, lending rates (i.e. cost of finance) and vacancy rates in the industrial property market. The focus of our study is to examine the key economic variables as well as supply-side parameters including zoning, natural restrictions and industrial land converted to other uses. The commonly used economic parameters are: GDP, industrial GVA, employment and property price (or rental price). Other factors determining the supply of land are out of the focus of the study.

In the equilibrium, demand is equal to supply and there is no incentive in the property market to change the activities of the economic agents. Solving the equilibrium of demand and supply equations of Eqs (1) and (2), a reduced form of new equation of developed industrial land area, LAND_i,t_ representing the conditions of demand and supply in the industrial property market, can be formed as follows:

$$LAND_{i,t}=f\left(P_{i,t},P_{i,t-s},R_{i,t},R_{i,t-s},E_{i,t},E_{i,t-s},A_{i,t},C_{i,t},Z_{i,t},N_{i,t},LAND_{i,t-s}\right)$$ (3)

As prices and rental prices are linked through capitalisation rates, there is a direct relationship between prices and rents in the market. Assuming that capitalisation rate remains unchanged, property price can be written as a function of rental price. However, in this study rental prices of Dutch industrial properties are not included in the analysis as the time-series of rental price data is unavailable at the regional level. As a future focus, we recommend rental prices to be considered in the analysis based on the availability of the subject data.

#### Estimation methodology

Based on construction demand forecasting literature, the process determining the variable of industrial land depends on the set of some related past variables in the same way as industrial land depend on the set of its past values. In our sample, data for all the related variables is specified at the regional level. In this, we can proceed to generate a dynamic panel data model considering the spatial variations in our pooled time-series data. Eq (3) can be adapted to a dynamic panel data model with the lagged effects of the dependent variable i.e. LAND_i,t-s_ incorporated in the error term (see Maddala and Lahiri [70] for details)

$$y_{it}=\gamma_{i}+\sum_{k=1}^{K}\alpha_{i}^{\left(k\right)}x_{i,t-k}+\varepsilon_{it},i=1,\ldots ,N;t=1,\ldots ,T$$ (4)

$$\varepsilon_{it}=\rho \varepsilon_{i,t-1}+\omega_{it},\left|\rho \right|$$

where y_it_ is the industrial land where the industrial construction is taking place, k represents the time lag, γ_i_ is the panel-level effect; x_i,t-k_ is a 1×k_1_ vector of exogenous variables for each individual region i and at time t; α_i_^(k)^ is k_1_×1 vector of parameters to be estimated; ω_i_ are I.I.D. error terms over the whole sample with a variance σ^2^_ε_. The γ_i_ and ω_i_ are assumed to be independent for each i over all t.

As the explanatory variables may be correlated with the unobserved panel-level effects, the estimation of Eq (4) by using standard OLS methods makes the estimators inconsistent. Following Arellano and Bond [71], Arellano and Bover [72], and Blundell and Bond [73], the above-mentioned issues have been covered through the application of Generalised Methods of Moments (GMM) approach. Consequently, the estimators are constructed by first differencing Eq (4) to remove panel-level effects and using instruments to form moment conditions. The first-differenced version of (4) can be written as:

$$\Delta y_{it}=\sum_{k=1}^{K}\alpha_{i}^{\left(k\right)}\Delta x_{i,t-k}+\Delta \varepsilon_{it},i=1,\ldots ,N;t=3,\ldots ,T$$ (5)

$$\Delta \varepsilon_{it}=\rho \Delta \varepsilon_{i,t-1}+\Delta \omega_{it},\left|\rho \right|<1$$

in which the panel-level effects are eliminated by the first differencing operation. The GMM approach utilises instruments for the estimation of Eq (5). The details on instrumental variables are provided in the Appendix.

### Data

The data used in the analysis consists of 40 regions at the NUTS3 (nomenclature of terrestrial units for statistics) level in the Netherlands, covering each year between 2000 and 2008. The NUTS3 coincide with the so-called COROP regions in the Netherlands, which has been originally designed to reduce cross-border commuting and provides a rough approximation of functional labour market regions in the Country [74]. Therefore, the NUTS3 level is selected as the spatial unit in the analysis considering that the NUTS3 level is the closest to the spatial structure of the Dutch labour and industrial land markets [18, 75]. The industrial land-use data was obtained from land-use maps for Netherlands for the years 2000, 2003, 2006, and 2008 provided by the PBL Netherlands Environmental Assessment Agency **(**http://www.pbl.nl/en/) and were utilised to compute the areas of industrial land, natural protection and naturally restricted areas for land development, and large-scale urban development zones which incorporates industrial land uses [76] for each corresponding year. The areas of industrial land, protected and naturally restricted areas, and industrial land in development zones for those years where data is unavailable were calculated through applying a linear interpolation between known data points for all regions. This was done to provide continuity in the time-series data regarding the areas of industrial land. The existing data points for the years 2000, 2003, 2006 and 2008 were individually checked for each NUTS 3 region, and a linear relationship was observed between the existing data points to a large extent.

The areas of industrial land converted to other land uses were computed through application of a GIS-based analysis. In this approach, two spatial datasets (vector datasets) for the two subsequent years were compared regarding the industrial land uses existing in period t-s and converted to other uses in the subsequent period t. It is noted that industrial uses were mostly converted to social and recreational activities as well as residential uses. From this analysis, the areas of industrial land converted to other uses were obtained for the years 2000, 2003, 2006 and 2008. The unknown data points between these years were developed through linear interpolation.

The accessibility indicators were obtained from the work of Jacobs-Crisioni et al. [77] where potential accessibility measures were computed throughout Europe for each municipality within the modelled country and for the NUTS2 regions outside of the modelled countries (see also Jacobs-Crisioni and Koomens [78]). The potential accessibility measure is computed based on the formula:

$$A_{i,t}=\sum_{i\neq j}^{m}P_{j,t}M_{ij,t}^{\gamma }$$

where A is the accessibility at origin municipality i; P is the population count in destination municipalities j in decade t; M represents the distance-decayed travel times in minutes; γ is assumed to be -1 as the real value of distance-decay is unknown for the study area. To compute the accessibility measures, domestic and foreign destinations (see Jacobs-Crisioni and Koomen [78] for the domestic and foreign accessibility component formulas) were taken into account; and the intra-zonal destinations were considered based on the Frost and Spence [109] approach in such a way that internal distance d_j_ is assumed to be $d_{j}=0.5\sqrt{AREA_{j}/\pi }$. The formula implies that intra-zonal distances are half of the radius of a hypothetically constructed circular zone. Travel times were obtained from the TRANS-TOOLS road network using the shortest path algorithm assuming free-flow travel times (for the details, we refer to Jacobs-Crisioni and Koomen [78]; Jacobs-Crisioni et al. [77]).

The accessibility measures computed for the Netherlands are originally provided at the municipality level and were aggregated to the NUTS3 level to be included in the current study. As the accessibility measures were computed for each of the decade between 1961 and 2011 [78], the values for the years from 2000 to 2008 were developed through applying a linear interpolation between 1991, 2001 and 2011.

This study utilises gross domestic product (GDP), sectorial gross value added (GVA aggregated for each of the industrial sectors considered in this study), sectorial employment (EMP-in industry) and industrial property price (PRICE) data for each NUTS3 in the Netherlands from the online database provided by Eurostat [79] and the Netherlands Bureau of Statistics [80]. Some basic statistics on economic parameters is summarised in Table 1 for each NUTS3 region.

**Table 1:** Descriptive statistics for the economic parameters.

Regions	Annually Added Industrial Land (in km2)		Annually Added Industrial GVA (in million Euro)		Annually Added GDP (in million Euro)		Annually Added Industrial Employment (in thousand)		Annually Added Industrial Property Price (in million Euro)	
	Mean	S. D.	Mean	S. D.	Mean	S. D.	Mean	S. D.	Mean	S. D.
1 East Groningen	10.34	0.08	540	17	2,700	200	9.79	0.65	1,980	450
2 Delfzijl	4.74	0.52	710	96	1,600	144	4.52	0.27	930	225
3 Rest of Groningen	16.49	1.01	7,300	2,760	17,000	3,810	19.87	1.70	7,290	1,370
4 North Friesland	16.16	1.63	1,700	185	8,800	883	16.34	1.08	4,980	1,330
5 South-West Friesland	5.92	0.63	360	41	2,300	258	6.64	0.26	1,600	502
6 South-East Friesland	10.98	0.95	810	73	4,800	552	13.82	0.29	3,000	861
7 North Drenthe	7.7	0.68	710	45	4,500	447	7.54	0.54	3,100	679
8 South East Drenthe	9.44	0.87	1,200	114	4,000	422	12.14	0.79	2,360	532
9 South West Drenthe	6.42	0.51	580	105	3,200	372	8.93	0.24	1,940	469
10 North Overijssel	18.08	1.73	1,800	288	10,000	1,250	23.18	0.61	6,150	1,710
11 South-West Overijssel	6.41	0.23	710	63	3,700	311	10.30	0.77	2,350	679
12 Twente	29.74	1.21	3,100	282	15,000	1,800	45.84	2.55	9,640	2,520
13 Veluwe	24.65	1.57	2,200	268	18,000	2,210	34.53	1.77	11,700	3,050
14 South-West Gelderland	15.47	0.83	1,900	138	9,100	1,150	22.96	10.08	3,710	1,080
15 Achterhoek	19.39	0.88	2,500	92	19,000	1,660	34.89	4.26	6,030	1,550
16 Arnhem & Nijmegen	25.26	0.89	830	83	5,800	737	24.19	11.10	11,000	3,100
17 Flevoland	16.85	1.48	850	190	8,200	1,450	13.11	1.54	5,750	2,070
18 Utrecht	36.12	2.09	3,700	291	43,000	4,500	46.24	5.49	23,900	6,570
19 Kop North Holland	12.75	0.82	740	55	8,000	854	12.54	1.49	5,410	1,520
20 Alkmaar	6.77	0.4	670	79	5,700	676	8.80	1.04	3,990	958
21 IJmond	11.76	0.31	1,600	323	5,000	693	15.01	1.68	3,270	555
22 Haarlem	3.69	0.05	520	87	5,200	336	6.81	1.25	3,840	1,040
23 Zaanstreek	6.45	0.16	860	113	3,700	336	9.44	1.22	2,390	586
24 Greater Amsterdam	39.71	2.76	3,600	279	57,000	7,080	43.53	6.34	36,100	10,300
25 Het Gooi-Vechtstreek	4.52	0.37	680	77	7,200	458	9.56	1.64	4,510	1,170
26 Leiden-Bollenstreek	10.67	1.01	1,400	149	10,000	1,070	16.52	1.96	6,960	1,490
27 The Hague	10.43	1.13	1,500	125	26,000	2,920	15.68	2.02	16,500	3,520
28 Delft-Westland	7.34	0.64	750	91	7,500	661	9.25	1.31	4,520	1,280
29 East-South Holland	11.7	0.89	960	61	8,300	703	13.75	1.75	5,220	1,560
30 Rijnmond	77.24	4.01	7,600	1,450	45,000	6,030	54.72	7.15	32,100	5,910
31 South-South Holland	18.44	0.88	1,800	164	11,000	1,070	24.49	3.14	6,150	1,260
32 Zeeuws-Vlaanderen	11.32	0.48	1,500	316	3,800	511	8.54	1.13	2,490	4,170
33 Overig Zeeland	17.38	1.54	1,300	314	6,600	909	14.79	1.80	4,860	1,370
34 West Brabant	43.5	2.28	5,700	421	20,000	2,110	46.03	5.81	13,500	3,710
35 Mid Brabant	23.58	1.4	2,000	124	12,000	1,330	27.61	3.74	8,250	2,320
36 North-East Brabant	30.71	1.42	3,800	404	19,000	2,050	47.39	5.89	11,600	2,910
37 South-East Brabant	35.43	1.96	4,500	706	22,000	2,630	67.07	8.32	15,100	4,270
38 North Limburg	17.58	1.65	1,400	80	7,600	750	23.06	2.95	5,480	1,280
39 Mid Limburg	13.57	0.8	1,600	212	6,000	801	18.30	2.45	4,020	9,830
40 South Limburg	28.55	0.97	3,800	224	18,000	1,500	39.60	6.30	10,900	2,290
Mean across Regions	18.08		1,994		12,413		22.18		7,870	
S. D. across Regions	1.07		233		1,423		2.10		8,100	
Min. of Region aggregate	16.46		1,724		10,345		17.30		604	
Max. of Region aggregate	19.61		2,401		14,633		24.69		48,700	

**Fig 1:**
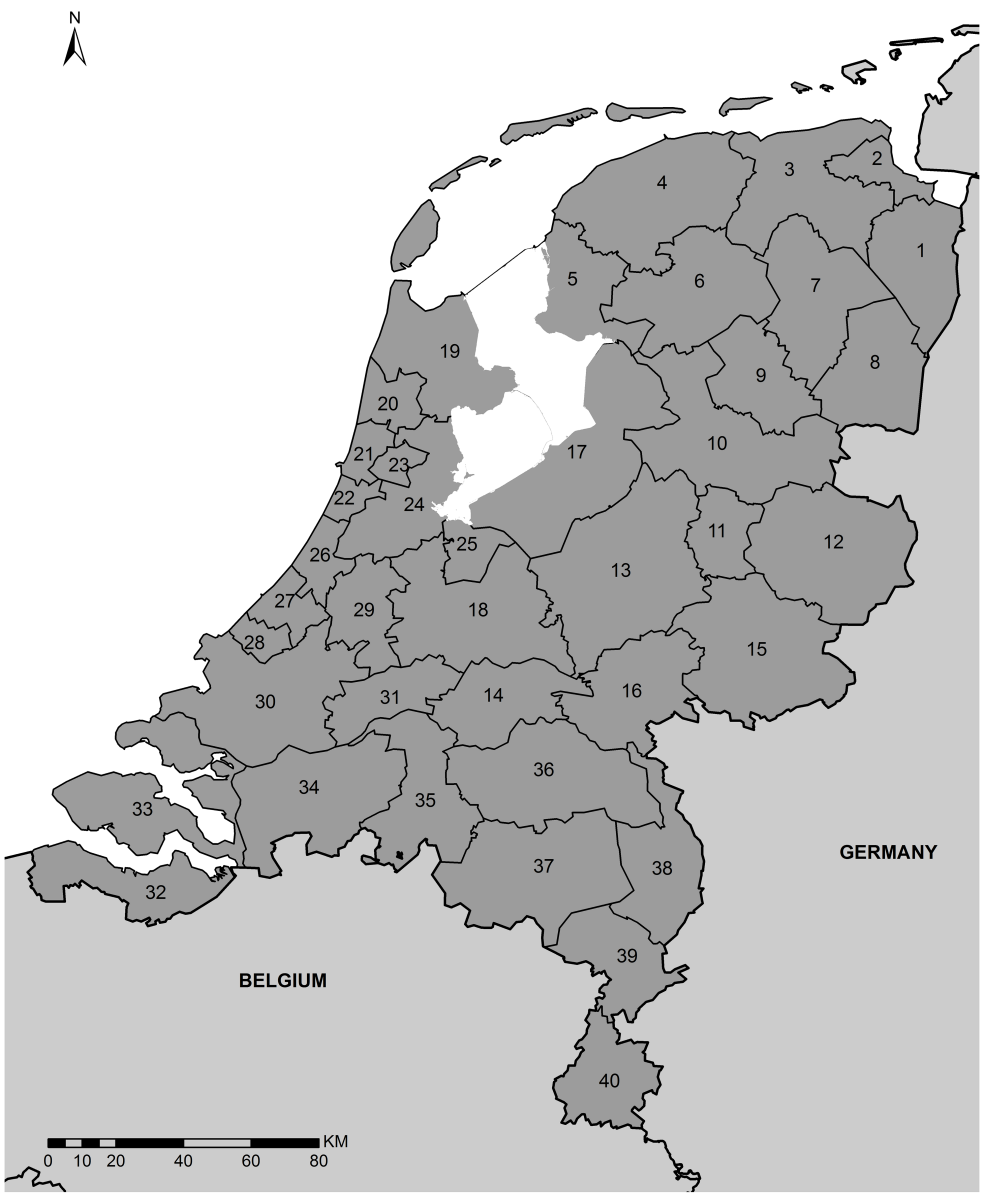
The study area.

From the table, it can be seen that there are considerable differences across regions concerning annual average values of GDP, industrial property price, industrial GVA, and employment. The highest values are observed in the regions of Greater-Amsterdam, Rijnmond, Utrecht, Rest of Groningen, and South-East Brabant, which are housing majority of the economic activities in the Netherlands. An overview of the study area and its regional sub-divisions can be seen in Fig 1.

### The Dutch industrial land market and land-use planning system

The lags between industrial demand and supply are sound in regions where there is scarcity of land for industrial development, strong regulations in real property markets, and strict institutional structures influencing the property transactions in the land markets. Netherlands is such an example of a region where there is scarcity of land ready-to-develop considering particularly the physical limitations in the Country (most of the land needs to be processed prior to development for the reasons of high water levels and low capacity of the soil to support the built structures in the country) strict land development restrictions imposed by various governmental layers, and a strict administrative system involving in various land-use planning activities. Land-use plans are highly restrictive in the Netherlands due to the fact that it is impossible to develop new urban areas without making changes in the land-use plans. Though land-use restrictions are important in the Country, it is also possible to have changes in the land-use plans. However, this requires a time consuming effort with the administrative procedures that may lead to uncertain outcomes.

With respect to the institutional structure, there are three levels of government to lay down a strategic plan. These consist of: ‘(a) the national spatial planning decisions, (b) the provincial regional plans, (c) the municipal structure plans’ [81]. While municipal structure plans are dictated by higher governmental levels, these structure plans prepared by the Dutch municipalities (complying with provincial and national plans) are the only legally binding and highly influential plans in determining the conditions in the land market (see Hajer and Zonneveld [81]; Needham and Louw [82] for a review of the institutional structure). The local land-use plans are also subject to a certain level of judicial flexibility and rarely overturned by higher planning authorities [30, 83]. The growth of industrial development is facilitated by large-scale plans to develop new industrial land uses, and the municipalities are the main responsible bodies for the supply of industrial land. In Needham and Louw’s ([82]: 84) explanation, “in the Netherlands, the public provision of industrial land is seen as a means of implementing spatial, economic and employment policy” (see also Badcock [84]; Louw et al.[30]).

Considering this highly restrictive land-use planning system, an increase in land values would be expected as the supply of land diminishes as a result of land-use restrictions. The evidence shows that its final effect on urban development is mostly negative [85]. However, it is also evident that ‘the spatial context and the competition between municipalities and jurisdictions can determine the outcomes of land-use regulation on urban development’ [85]. Spatial competition for urban jurisdictions is sound in the Netherlands, where spatial planning is characterised by ‘compact development’ policies resulting high development densities and ‘clustered de-concentration’ aiming at concentrating suburbanisation at specific locations to increase urban densities in those particular locations [74, 86].

While keeping high densities through successful management of urban development, the restrictive planning system has also led to successful open space preservation in the Country in the last five decades [87]. Nature conservation policies comprising, NATURA 2000-a European network of protected areas in the EU, and National Ecological Network (NEN)-a network of natural reserves areas put restriction on urban development to avoid conversion of protected land [88]. Koomen and Dekkers [89] suggest that there are more restrictive Buffer zone and Green Heart policies in the Netherlands, which cover important national landscapes as some of them were approved as world heritage sites by UNESCO. Regarding the preservation of Buffer zone and Green Heart, Koomen et al. [86] showed that between 1995 and 2003, the propensity of transformation from open space to urban use was significantly lower in these protected zones compared to other parts of the Randstad Area. Concerning the success in the application of open space preservation policies, a variable, namely natural limitations (see Eq 2 in the methodology section), is included in the supply equation to account for the impact of natural restrictions on urban development at the regional level. This variable represents the areas of natural protection zones such as NATURA2000 and NEN, and other natural restrictions for urban development such as water bodies and sludge areas in each NUTS3 region.

Besides open space preservation, large-scale urban development zones [76] introduced by the national spatial planning authorities in the Netherlands are highly successful in facilitating urban growth as these neighbourhoods also incorporate industrial and business areas aiming at reducing the commuting distance between new neighbourhoods and employment centres [85, 90]. Though these large-scale plans were introduced by national government, they are mostly implemented at the regional and particularly municipal level with the involvement of private parties to invest in these new urban development schemes. Therefore, municipalities compete with each other to attract residents and developers of industrial and business areas to their territories [90]. There is not much difference among the regional authorities and the municipalities in the application of spatial plans. It is mentioned in Broitman and Koomen [87] that ‘the previous national planning report has provided more freedom to regional and local authorities to meet their objectives’ (they referred to: VROM et al. [91]). The industrial land in each of these large-scale development zones can be considered as a planning restriction that should be implemented by the corresponding local authorities. Therefore, we use industrial land area in each urban development zone for each corresponding NUTS3 region in the study area (see Eq 2) to represent the spatial planning policies of the government authorities implemented at the regional and municipal levels (see Levkovich and Rouwendal [85]; Broitman and Koomen [87]) for the inclusion of spatial planning policies in the models of the land markets in the Netherlands).

**Fig 2:**
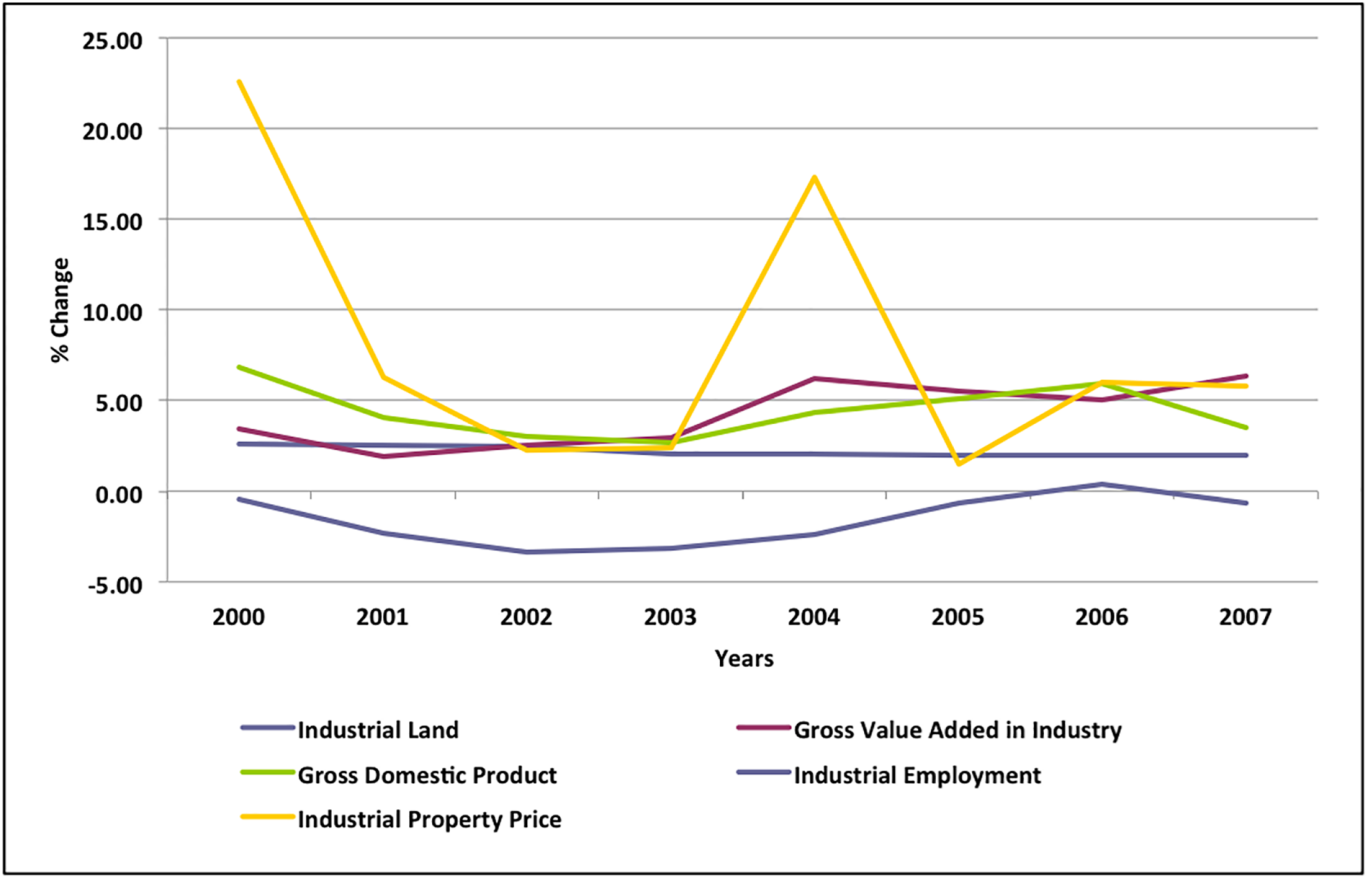
Percentage changes of industrial land, GVA (industry), GDP, industrial employment and industrial land price for Netherlands, 2000–2008. **Note:** Data on industrial land exists only for 2000, 2003, 2006, and 2008. The data for the remaining periods was interpolated using a linear relationship with the existing data.

In the Netherlands, there is a large gap between development of a land-use plan and provision of the industrial land for development [30]. Given this strict institutional and regulatory structure, municipalities have often developed their own methods of forecasting the demand for industrial land for the provision of serviced land without delay. Considering the competition among the municipalities in attracting higher numbers of firms than their neighbours, the supply of industrial land is higher than the sale/lease of land on industrial estates in the Netherlands [83]. The over-supply of industrial land can also be explained by the existence of time lags between the industrial land use and economic parameters, and an asymmetric relationship between land development and economic change. This can be observed from our data on the percentage changes of industrial land, GDP, GVA, property price and employment in the industrial sectors (Fig 2). From Fig 2, it is clear that developments in the industrial land respond slowly to the fluctuations in the economic parameters. For instance, there are considerable changes in GDP, GVA, industrial property price and the number of industrial employment between 2000 and 2008, which are associated with modest changes in the industrial land developments.

Considering the discussed characteristics of the industrial land market, long-term forecasts of industrial land use have become an important issue in the Netherlands. Therefore, various methods are used in Netherlands as well as other countries for the estimation of future industrial land use (for detailed review, see Beckers and Schuur [18]). Knoben and Traa [92] (cited in: Beckers and Schuur [18]) stated that employment-based approach is the most commonly used methodology in Netherlands among others. A similar approach based on employment forecasts is also applied in European and non-European countries such as Ireland, UK, and Australia [93, 94]. This methodology is based on future projection of employment by sector and multiplying these numbers with a land-use parameter to get the average size of land requirements per worker [18, 94]. There are also other variables utilised in forecasting of industrial land such as economic output and income [10] and real property values [16]. The influence of a set of common economic fundamentals on the value of real property can be found in Hendershott et al. [95] and Wilson and Zurbruegg [96] (see Beckers and Schuur [18] for a detailed review of industrial land forecasting methods and the use of employment-based method in the Netherlands). Considering the importance of key economic parameters in forecasting industrial land in Netherlands and internationally, this study focuses on the relationship between industrial land development and these indicators to uncover the influence of lags of the subject indicators on the development of industrial land use.

## Estimation results

### Results from panel unit root tests

Pooled time-series data tend to exhibit a time trend implying non-stationary data processes due to the existence of time dependency across means, variances and co-variances of the variables. Therefore, research on testing for unit roots in panels have been developed [97, 98]. It is demonstrated in the literature that the application of regression analysis to non-stationary data results in misspecification errors and misleading statistics such as high R^2^’s and t-statistics [99]. Therefore, it is important to test stationary of the data prior to regression applications. To this end, we applied three different tests in order to test the stationary feature of our dynamic panel data. These consist of: Levin, Lin and Chu (LLC) test, Fisher-Dickey-Fuller (FDF) test, and Im, Pesaran and Shin (IPS) test. The first test assumes that there is a common unit root process across the cross sections. This follows an Augmented Dickey Fuller (ADF) specification to test the hypothesis of a unit root against the alternative that each time series is stationary [100]:

$$\Delta y_{it}=\rho y_{i,t-1}+\sum_{j=1}^{pi}\theta_{ij}\Delta y_{i,t-j}+\delta_{mi}x_{mt}+\varepsilon_{it},m=1,2,3$$ (6)

where y_it_ refers to the pooled variable, x_mt_ represents exogenous variables in the model (i.e. individual time trends, spatial fixed effects), *δ*_*mi*_ are the corresponding coefficients, p_i_ is the lag order, and *ε*_*it*_ are the error terms which are assumed to be mutually independent. By contrast, Fisher-Dickey-Fuller, and Im, Pesaran and Shin tests estimate a separate ADF regression for each of the cross sections to allow for individual unit root processes across the cross sections.

Table 2 summarises the results from unit root tests on the subject variables embedded in the model. The variables were tested by using both the first and the second lag specifications (Eq 6), and the first differences of the variables were also tested. The results from these tests suggest that except the variable LAND, most of the tests concerning other variables fails to reject the existence of a unit root following the use of the first lag specifications. The first differences of some variables become stationary as reported by the LLC, Fisher-DF and IPS tests; however, this is not the case for the LAND variable. Regarding LAND, both the first and the second lag specifications rejects the null hypothesis across all three tests implying a stationary process of the panel data. Therefore, we employ the LAND variable in the model without considering its first difference as it does not follow a stationary process. Since the results from the three test statistics suggest that GVA, GDP, PRICE and EMP should be taken by following the second lag (as it is stationary by contrast to the first lag specifications), we continue our analysis by using the second lag specifications of the subject variables in the model.

**Table 2:** Panel unit root tests.

Variable	Method	Statistic	Stat-value	P-value
LAND (_1)	LLC	Adj. t*	-12.92	0.0001***
	Fisher-DF	Inv. Chi-squared	115.56	0.0057***
	Im-Pesaran-Shin	w-t bar	-1.48	0.0685**
LAND (_2)	LLC	Adj. t*	-45.59	0.0001***
	Fisher-DF	Inv. Chi-squared	206.06	0.0001***
	Im-Pesaran-Shin	w-t bar	-0.01	0.0001***
ΔLAND	LLC	Adj. t*	-1.11	0.1316
	Fisher-DF	Inv. Chi-squared	97.78	0.0861**
	Im-Pesaran-Shin	w-t bar	3.03	0.9988
GVA (_1)	LLC	Adj. t*	-3.80	0.0001***
	Fisher-DF	Inv. Chi-squared	54.04	0.9885
	Im-Pesaran-Shin	w-t bar	4.13	0.9998
GVA (_2)	LLC	Adj. t*	-29.83	0.0001***
	Fisher-DF	Inv. Chi-squared	352.98	0.0001***
	Im-Pesaran-Shin	w-t bar	-2.03	0.0211***
ΔGVA	LLC	Adj. t*	-11.47	0.0001***
	Fisher-DF	Inv. Chi-squared	254.06	0.0001***
	Im-Pesaran-Shin	w-t bar	-2.59	0.0047***
GDP (_1)	LLC	Adj. t*	-12.51	0.0001***
	Fisher-DF	Inv. Chi-squared	148.57	0.0001***
	Im-Pesaran-Shin	w-t bar	0.80	0.7900
GDP (_2)	LLC	Adj. t*	-45.77	0.0001***
	Fisher-DF	Inv. Chi-squared	432.53	0.0001***
	Im-Pesaran-Shin	w-t bar	-9.96	0.0001***
ΔGDP	LLC	Adj. t*	-10.71	0.0001***
	Fisher-DF	Inv. Chi-squared	98.42	0.0794**
	Im-Pesaran-Shin	w-t bar	0.14	0.5563
EMP (_1)	LLC	Adj. t*	3.60	0.9998
	Fisher-DF	Inv. Chi-squared	211.82	0.0001***
	Im-Pesaran-Shin	w-t bar	-0.70	0.2408
EMP (_2)	LLC	Adj. t*	-6.05	0.0001***
	Fisher-DF	Inv. Chi-squared	330.14	0.0001***
	Im-Pesaran-Shin	w-t bar	-9.80	0.0001***
ΔEMP	LLC	Adj. t*	-9.12	0.0001***
	Fisher-DF	Inv. Chi-squared	251.01	0.0001***
	Im-Pesaran-Shin	w-t bar	-2.15	0.0157**
PRICE (_1)	LLC	Adj. t*	-24.55	0.0000***
	Fisher-DF	Inv. Chi-squared	374.22	0.0000***
	Im-Pesaran-Shin	w-t bar	-7.77	0.0000***
PRICE (_2)	LLC	Adj. t*	-16.91	0.0001***
	Fisher-DF	Inv. Chi-squared	274.8	0.0001***
	Im-Pesaran-Shin	w-t bar	-5.03	0.0001***
ΔPRICE	LLC	Adj. t*	-13.36	0.0000***
	Fisher-DF	Inv. Chi-squared	489.85	0.0000***
	Im-Pesaran-Shin	w-t bar	-8.98	0.0000***

*Note*: Δ is the first order difference;

***significant at 1% level;

**significant at 5% level;

*significant at 10% level;

(_1) and (_2) refer to the first and second lag specifications to be used in the regressions from which panel unit root test statistics are computed.

### Results from dynamic panel regressions

Regression analysis was conducted to estimate Eq (5) applying GMM. Prior to the regression analysis, partial correlation coefficients across explanatory variables were computed for the pooled data and those having significant correlation coefficients are presented in Table 3. Considering the collinearity between ‘GDP’ and ‘GVA’, GVA to GDP ratio was calculated and included in the analysis as an additional variable. There is significant collinearity between pairs of some other variables, which are also presented in the Table. These are various combinations of the variables including: ‘ACCESS’, ‘EMP’, ‘PRICE’ and ‘CONV_LAND’. Therefore, these variables were included in the regressions either separately or they were treated with caution.

**Table 3:** Correlations among economic and land-use parameters (pooled data).

Variables	Correlation Coefficients	Variables	Correlation Coefficients
GVA and GDP	0.798**	ACCESS and EMP	0.823**
EMP and GDP	0.718**	ACCESS and PRICE	0.816**
EMP and GVA	0.731**	CONV_LAND and EMP	0.751**
PRICE and GDP	0.962**	CONV_LAND and PRICE	0.637**
PRICE and EMP	0.709**	CONV_LAND and ACCESS	0.689**
PRICE and GVA	0.652**		

**Statistical significance at the 5% level

We have specified industrial land development as a log-linear function of exogenous variables as defined in Eq (3). In order to compare the impact of lagged values of predictors with the contemporaneous values, we estimated two sets of model specifications given as

$$\text{Models }1:\;LAND=f\left(Z_{k},X_{k}\right);k=0$$ (7)

$$\text{Models }2:\;LAND=f\left(Z_{k},X_{k}\right);k=0;2;4;6;8$$ (8)

Where ‘X’ stands for economic parameters, ‘Z’ are the other parameters and ‘k’ represents the time lags as described previously. Tables 4 and 5 present the results for GMM system equations and the associated diagnostic test statistics for various combinations of variables selected as instruments. The tables specifically demonstrate the results concerning the regressions which do not show any misspecification errors following the diagnostic test statistics. From Tables 4 and 5, it can be noted that Eq (3) representing both supply and demand conditions is estimated in regressions B1, C1, B2, C2 and reduced forms of the equation are also estimated as presented in the related Tables. For instance, regressions A1 and A2 represent estimations of the demand equation of Eq (1) while regressions D1 and D2 refer to the supply equation of Eq (2).

**Table 4:** Estimation results for contemporaneous variables (Models 1).

Variable	Regr. (A1)	Regr. (B1)	Regr. (C1)	Regr. (D1)
	GMM-INST.1	GMM-INST.2	GMM-INST.3	GMM-INST.4
Log GVA	0.581** (0.109)			
Log EMP	0.058** (0.023)	0.348** (0.169)		
Log ACCESS	0.177** (0.063)		0.385** (0.049)	
Log GVA/GDP		0.367** (0.165)	0.554** (0.235)	
Log PRICE		0.456** (0.106)		0.557** (0.082)
Log ZONING		0.005** (0.001)	0.007** (0.005)	0.006 (0.007)
Log NAT_REST		-0.026 (0.032)	-0.017* (0.062)	-0.044 (0.129)
Log CONV_LAND		0.007 (0.036)	-0.002 (0.023)	0.098** (0.023)
Constant	-5.943** (0.629)	-4.346** (0.524)	-1.568** (0.589)	-4.007** (0.865)
Number of observations	440	440	440	440
Number of groups	40	40	40	40
Wald Chi (2) statistic	302.56**	272.42**	112.34**	192.32**
*Diagnostic Test Statistics*				
Arellano-Bond statistic (1)	-1.679[0.093]	-3.152 [0.002]	2.735 [0.006]	2.318 [0.02]
Arellano-Bond statistic (2)	1.523 [0.127]	-1.335 [0.182]	1.887 [0.060]	1.811 [0.07]

Notes:

**Statistical significance at the 5% level;

* significance at the 1% level.

In parenthesis are the robust standard errors.

INST.1, INST.2, INST.3 and INST.4 refer to instrumental variable sets for the first difference equations. The two sets differ by including a number of different variables. The details are given below:

INST.1: Diff. Eq. Industrial Land (-2); Level Eq. Price INST.2: Diff. Eq. Industrial Land (-2); Level Eq. Mining, Rail, Airport, GVA INST.3: Diff. Eq. Industrial Land (-2); Level Eq. Mining, GVA INST.4: Diff. Eq. Industrial Land (-2), Access; Level Eq. Mining, Rail, Access

ACCESS: accessibility; ZONING: industrial land area in development zones; NAT_REST: natural protection and naturally restricted areas for development; CONV_LAND: industrial land converted to other land use

**Table 5:** Estimation results for contemporaneous and lagged variables (Models 2).

Variable	Regr. (A2)	Regr. (B2)	Regr. (C2)	Regr. (D2)
	GMM-INST.5	GMM-INST.6	GMM-INST.6	GMM-INST.7
Log ACCESS	0.142**(0.037)		0.159** (0.030)	
Log ZONING		0.001** (0.001)	0.003** (0.001)	0.0123** (0.002)
Log NAT_REST		-0.045** (0.011)	0.429** (0.031)	-0.293** (0.093)
Log CONV_LAND			-0.012** (0.003)	-0.007* (0.015)
Log GVA	0.008** (0.028)			
Log GVA (-2)	0.053* (0.040)			
Log GVA (-4)	0.118** (0.042)			
Log GVA (-6)	0.138** (0.035)			
Log GVA (-8)	0.136** (0.010)			
Log EMP	0.097 (0.043)	0.021 (0.071)		
Log EMP (-2)	0.039** (0.031)	0.059** (0.025)		
Log EMP (-4)	0.084* (0.051)	0.206** (0.054)		
Log EMP (-6)	0.040** (0.015)	0.115** (0.025)		
Log EMP (-8)				
Log GVA/GDP		0.027 (0.056)	0.102 (0.043)	
Log GVA/GDP (-2)		0.086* (0.065)	-0.072 (0.068)	
Log GVA/GDP (-4)		0.199** (0.060)	0.113* (0.068)	
Log GVA/GDP (-6)		0.307** (0.063)	0.072** (0.056)	
Log GVA/GDP (-8)		0.245** (0.055)	0.069** (0.051)	
Log PRICE		0.415** (0.101)		1.734** (0.503)
Log PRICE (-2)		-0.246**(0.115)		0.085** (0.335)
Log PRICE (-4)		0.326** (0.141)		0.837** (0.360)
Log PRICE (-6)		0.147** (0.058)		0.862** (0.239)
Log PRICE (-8)				-1.418**(0.299)
Constant	-4.331** (0.434)	-6.002**(0.375)	-1.450** (0.140)	-9.401** (0.962)
Number of observations	120	120	120	120
Number of groups	40	40	40	40
Wald Chi (2) statistic	12052.44**	56930.24**	4930.51**	468.23**
*Diagnostic Test Statistics*				
Sargan-Chi (2) statistic	15.902 [0.998]	70.750[0.322]	99.952[0.101]	71.916[0.199]

Notes:

**Statistical significance at the 5% level;

* significance at the 1% level.

In parenthesis are the standard errors.

INST.5 to INST.7 refer to instrumental variable sets for the first difference equations. The three sets differ by including a number of different variables. The details are given below:

INST. 5: Diff. Eq. Industrial Land (-2); Price (-2); Mining (-2); Public Facilities (-2); Conv_Land; Level Eq. Mining (-2); Conv_Land INST. 6: Diff. Eq. Industrial Land (-2); Mining (-2); Rail (-2); Airport (-2); Conv_Land; Access; Level Eq. Mining (-2); Land_Conv; Access INST. 7: Diff. Eq. Industrial Land (-2); GDP; Level Eq. Mining (-2)

We first discuss the selection of instrumental variables utilised in the estimations of Models 1 and 2 based on various combinations of explanatory variables. Basically, an instrumental variable is a variable that is uncorrelated with the error term but correlated with the explanatory variables in the estimated model [101]. A set of instrumental variables, which is correlated with the explanatory variables in Models 1 and 2, but uncorrelated with the error term in our dynamic panel data model was developed. These include the variables representing ‘areas of mining sites, public facilities, airport and rail infrastructure’, ‘areas of land converted from industry to other uses’, ‘GDP’, ‘accessibility’, ‘price’, and ‘population’ which were computed for each NUTS3 region within the period 1996–2008. The areas of mining sites, public facilities, and airport and rail infrastructure were derived from the land-use maps for Netherlands for the years 1996, 2000, 2003, 2006, and 2008. The data for missing years were linearly interpolated by using the known data points for all the NUTS3 regions.

The above outlined instrumental variables can represent area, industry and location specific characteristics in our study area. This can be explained, for instance, by the existence of a metropolitan or an urban area in a region implying that the region accommodates large number of population, highly skilled labour force and intensive physical infrastructure, directly linked with the growth of industrial properties.

In the first difference equations regarding Models 1 and 2, we consider either two-period lagged values of the variables including mining sites, airport, rail, public facilities, price, population; one-period lagged values of accessibility, areas of land converted from industry to other uses, GDP, and/or the dependent variable i.e. LAND were lagged two periods, as potential instruments. This points to the assumption of weak exogeneity (Eq A2 in Appendix) concerning all exogenous variables. From the Sargan statistics given at the bottom of Table 5, the weak exogeneity of the different variable sets (see the Table notes) is not rejected for all the regressions estimated for various combinations of the explanatory variables. These test results indicate that there are no misspecification errors in the selection and the use of the specified instruments in the estimations of Models 2.

Table 4 reports the Arellano-Bond statistics to detect zero first-order and second-order correlations in the level residuals. The moment conditions (Eq A1 in Appendix) set for the estimation of dynamic panel model utilise the orthogonality conditions between the differenced errors and the lagged values of the dependent variable [100]. This is based on the assumption that the error terms in our dynamic panel model (Eq 5) are serially uncorrelated and the differenced error is with unit root [100]. These conditions are satisfied if the null hypothesis of the absence of first-order serial correlation is rejected while the null of the absence of second-order serial correlation is not rejected. In Table 4, we present the regressions which satisfy the stated conditions.

From Table 4, almost all the explanatory variables have expected signs except CONV_LAND, and most of them are significant at the 1% level. The sign of the CONV_LAND variable is ambiguous as it has a positive sign in the regressions B1 and D1 and a negative sign in C1. It may stem from possible correlations between CONV_LAND and other explanatory variables included in the regressions. Land zoning policies and accessibility have a positive impact while natural restrictions for development have an expected negative impact on industrial land developments. Regarding estimations of Model 1, the coefficients for economic output, output-to-income ratio and price are larger than other predictors. For instance, the positive sign of GVA/GDP implies that high share of industrial GVA in national income results in an increase in industrial space. The B1 outcomes show that variations in industrial demand in Netherlands are mainly explained by price that is followed by manufacturing output to income ratio and then employment. When we exclude economic income and output from the regressions as in D1, industrial property price is considered as highly influential in explaining industrial land developments. The coefficients indicate the elasticity of estimated variables on the industrial land development, that is, a 1% increase in industrial employment corresponds with 0.05% increase in land use in Model A1, and with 0.34% in Model B1.

The second model specifications in Table 5 explain the impact of lagged economic predictors on industrial land use. We keep all the contemporaneous predictors the same as specified in the first group of models (Table 4) and added different lags of economic parameters to the corresponding models (see Eq 3) as shown in Table 5. From A2, B2 and C2 it is followed that the contemporaneous value of EMP and GVA/GDP becomes insignificant in contrast to Models A1, B1 and C1 (Table 4) when the lagged values of these predictors are added to the regressions. This points to the existence of development lags in the industrial property market when there is an upturn (or down turn) in the economy. The increase in economic activity results in an increase in user demand. However, firms tend to adjust the existing space according to the requirements of new production activities as suggested by Ball et al. [49]. Additional demand is absorbed by the vacant industrial properties in the short run and this will result in increasing land values and property prices. This may encourage developers to initiate new industrial property investments. Considering development constraints and long time period required for the delivery of the new construction projects, there is a lag between industrial land development and the growth in economic activities [25, 49]. The insignificant coefficients of EMP and GVA/GDP confirm this fact. On the other hand, from A2 and B2, we observe an increasing influence of the lagged GVA on industrial land. The coefficients of GVA and GVA/GDP with two-period to eighth-period lags lie in the range of 0.008 to 0.13 in Model A2 and 0.027 to 0.30 in B2, respectively; the sixth-period lag indicating a strong reinforcing effect on the dependent variable in both models.

To analyse the possible impact of employment on industrial land, a separate model with current and lagged values of employment variable was also estimated. The coefficients of this model were found insignificant and did not satisfy the model specification test statistics addressing the bias in the estimated coefficients. Therefore, we estimated employment variable with the other variables to have robust estimates for the subject variable. The outcomes of these models indicate that the coefficients of EMP are quite significant for the two, four and six-period lags where the four-period lags in A2 and B2 demonstrate the highest elasticity on industrial land developments.

As can be followed from Table 5, most of the explanatory variables in the regressions have the expected signs with significant coefficients. Accessibility and land zoning policies have positive impacts on industrial land developments as can be followed from the Models presented in the Table. By contrast, A2 and C2 indicate insignificant coefficients for either contemporaneous value of EMP and GVA/GDP or second-period lag of GVA/GDP, which has an unexpected negative coefficient in the latter case. Natural restrictions have an expected negative impact on industrial land development in Models B2 and D2. However, it has an unexpected positive impact in Model C2. From C2 and D2, we note that the amount of industrial land converted to other uses has a negative impact on industrial land. The models incorporating PRICE in the regressions show that its current value has the largest impact on industrial land which is followed by four-period and six-period lags as highlighted by Models B2 and D2. This confirms the fact that property developers generally make investment decisions based on the prices at the time of delivery as well as the prices in the recent past periods [42, 69].

To demonstrate the individual effect of GVA, EMP and PRICE on industrial space, we estimated various dynamic panel regressions for individual lags of the subject variables on industrial land. The results from these regressions are provided in Tables 6, 7 and 8. From Table 6, we observe a decreasing influence of GVA from two-period lag to the eight-period lag; two-period and four-period lags having the highest impact on industrial land developments indicating the existence of lags between economic growth and land development in Netherlands. A similar pattern is observed with PRICE (Table 8) where the coefficients range from 0.15 to 0.34; the contemporaneous value of the variable having the strongest impact on industrial land. This coincides with the results presented in Table 5. As suggested by the theory, developers take into account the property price information in the very recent periods for their new investment decisions. Therefore, we suggest use of the contemporaneous value of PRICE as an indicator for the developers in the market regarding new industrial property investments. By contrast to previous findings, EMP (Table 7) has an increasing effect on industrial land, the eight-period lag indicating a strong reinforcing effect on the dependant variable. This implies that firms initially do not demand for new industrial land following an increase in number of employment but they prefer adjusting their existing industrial space to accommodate new workers. If there is excess demand for new space following the initial adjustments, firms demand for additional industrial land in the long run. The results in Table 7 demonstrate existence of lags between growth in employment and land development in the long run. These issues should be taken into account in the industrial land forecasting work which utilises employment-based methods as in the case of Netherlands.

**Table 6:** Estimation results for individual variables: GVA.

Variable	Lag: 0	Lag: 2	Lag: 4	Lag: 6	Lag: 8
	GMM-Robust	GMM-Robust	GMM-Robust	GMM-Robust	GMM-Robust
LogGVA	0.343**(0.108)				
Log GVA (-2)		0.387**(0.039)			
Log GVA (-4)			0.359**(0.029)		
Log GVA (-6)				0.278**(0.026)	
Log GVA (-8)					0.143**(0.026)
Constant	-2.001** (0.989)	-2.382** (0.359)	-2.113**(0.273)	-1.366** (0.241)	-0.124**(0.237)
Number of observations	440	440	440	440	440
Number of groups	40	40	40	40	40
Wald Chi (2) statistic	10.07**	96.94**	143.87**	110.49**	30.22**
*Diagnostic Test Statistics*					
Sargan-Chi (2) statistic	12.67[0.999]	126.66 [0.765]	119.05[0.818]	86.82[0.942]	68.06[0.942]

Notes:

**Statistical significance at the 5% level.

In parenthesis are the standard errors.

GMM-Robust: Diff. Eq. Industrial Land (-2); Mining(-2), Airport(-2), Public Facilities(-2), Population(-2), all differences are forward-orthogonal deviations.

**Table 7:** Estimation results for individual variables: EMP.

Variable	Lag: 0	Lag: 2	Lag: 4	Lag: 6	Lag: 8
	GMM-Robust	GMM-Robust	GMM-Robust	GMM-Robust	GMM-Robust
Log EMP	-0.219**(0.202)				
Log EMP (-2)		0.115**(0.026)			
Log EMP (-4)			0.164**(0.017)		
Log EMP (-6)				0.112**(0.012)	
Log EMP (-8)					0.658**(0.113)
Constant	2.065** (0.854)	0.666** (0.110)	0.469**(0.073)	0.698** (0.241)	-0.544**(0.469)
Number of observations	440	360	280	200	120
Number of groups	40	40	40	40	40
Wald Chi (2) statistic	1.18	19.23**	90.20**	88.06**	33.72**
*Diagnostic Test Statistics*					
Sargan-Chi (2) statistic	2.204[0.999]	142.62 [0.399]	143.12[0.279]	111.91[0.405]	67.60[0.355]

Notes:

**Statistical significance at the 5% level.

In parenthesis are the standard errors.

GMM-Robust: Diff. Eq. Industrial Land (-2); Mining(-2), Airport(-2), Public Facilities(-2), Population(-2), all differences are forward-orthogonal deviations.

**Table 8:** Estimation results for individual variables: PRICE.

Variable	Lag: 0	Lag: 2	Lag: 4	Lag: 6	Lag: 8
	GMM-Robust	GMM-Robust	GMM-Robust	GMM-Robust	GMM-Robust
Log PRICE	0.343**(0.069)				
Log PRICE (-2)		0.250**(0.012)			
Log PRICE (-4)			0.208**(0.010)		
Log PRICE (-6)				0.152**(0.008)	
Log PRICE (-8)					0.180**(0.017)
Constant	-2.205** (0.675)	-1.275** (0.119)	- 0.852**(0.100)	-0.299** (0.086)	-0.548**(0.167)
Number of observations	440	360	280	200	120
Number of groups	40	40	40	40	40
Wald Chi (2) statistic	24.52**	413.82**	398.86**	291.58**	106.56**
*Diagnostic Test Statistics*					
Sargan-Chi (2) statistic	4.465[0.999]	96.61 [0.997]	115.05[0.880]	93.14[0.861]	58.85[0.658]

**Statistical significance at the 5% level.

In parenthesis are the standard errors.

GMM-Robust: Diff. Eq. Industrial Land (-2); Mining(-2), Airport(-2), Public Facilities(-2), Population(-2), all differences are forward-orthogonal deviations.

## Evaluation of forecasting accuracy

The forecasts generated by Models 1 and 2 were evaluated on three measures of accuracy, namely, the average absolute error (AAE), the total absolute error (TAE), and the relative difference (RD). These measures can be explained as follows.

Average absolute error

$$AAE=\frac{\sum_{i}\left|E_{i}-A_{i}\right|}{n}$$

Total absolute error

$$TAE=\left(\frac{\sum_{i}\left|E_{i}-A_{i}\right|}{\sum_{i}A_{i}}\right)\times 100$$

Relative difference

$$RD=\left(\left(\frac{\sum_{i}E_{i}}{\sum_{i}A_{i}}\right)-1\right)\times 100$$

Where E_i_ is the estimated industrial land, A_i_ is the actual industrial land at region i; and n is the total number of NUTS3 regions. Using these measures, we can compare the forecasting accuracy of the models for the years 2000, 2003, 2006 and 2008 for the first group of models, and 2006 to 2008 for the second group. The results obtained for the models are shown in Tables 9 and 10.

**Table 9:** Measures of accuracy for out-of-sample forecasts for the first group models.

Year	Model	TAE (%)	RD (%)	AAE
**2000**	**A1**	10.11	0.29	0.11
	**B1**	10.03	-4.31	0.11
	**C1**	12.82	2.21	0.14
	**D1**	12.68	-2.57	0.14
**2003**	**A1**	9.27	-0.42	0.11
	**B1**	8.15	0.54	0.09
	**C1**	11.90	-1.45	0.14
	**D1**	10.80	0.96	0.12
**2006**	**A1**	9.77	-0.86	0.12
	**B1**	8.43	0.58	0.09
	**C1**	12.43	-2.98	0.15
	**D1**	12.10	0.28	0.14
**2008**	**A1**	8.79	-0.05	0.10
	**B1**	8.09	1.25	0.09
	**C1**	11.85	-3.59	0.14
	**D1**	12.68	0.34	0.15

Note: AAE is given in logarithmic value of industrial land in km^2^

**Table 10:** Measures of accuracy for out-of-sample forecasts for the second group models.

Year	Model	TAE (%)	RD (%)	AAE
**2006**	**A2**	8.08	-0.07	0.09
	**B2**	10.12	0.07	0.11
	**C2**	10.62	0.026	0.12
	**D2**	15.32	-0.81	0.18
**2007**	**A2**	8.23	-0.03	0.09
	**B2**	9.80	-0.02	0.11
	**C2**	10.64	0.00	0.12
	**D2**	15.83	-0.21	0.18
**2008**	**A2**	8.26	-0.05	0.09
	**B2**	9.80	-0.05	0.11
	**C2**	10.41	-0.02	0.12
	**D2**	15.44	-0.58	0.18

Note: AAE is given in logarithmic value of industrial land in km^2^

Results in Table 9 show that Model A1 which utilises contemporaneous values of EMP, GVA and ACCESS, and Model B1 which incorporates supply conditions in addition to parameters representing demand, performed better than other models. Regarding Models A1 and B1, the TAE is within the limit of around 10%, which is generally considered as acceptable [65] and the AAE is the smallest among all others. For the year 2000, Model A1 overestimates while B1 underestimates total industrial land in Netherlands. In 2003, 2006 and 2008, Model A1 underestimates while B1 overestimates industrial land. The underestimations of the Models fall in a range from -0.05 to -4.31 and overestimations range from 0.29 to 1.25.

From Table 10, it is observed that Model A2 is the best performer considering the smallest values of TAE and AAE among others and also showing one of the lowest relative differences. Models B2 and C2 perform better than Model D2 considering the TAE measures for B2 and C2 are consistently within the acceptable limit of 10% in contrast to the TAE for D2 which is more than 15%. This indicates that the model incorporating supply-side parameters as the only driver for land-use change is not able to perform better than other models which consider other economic variables in the predictions. The inclusion of all economic parameters to the predictions as in Model B2 (that is embedding both supply and demand conditions) and the inclusion of demand conditions as in Model A2 improves the prediction accuracy as the Models perform better than others.

Finally, when we compare the results in Tables 9 and 10, it can be concluded that the inclusion of the lagged values of the predictors (as in Models 2) to the contemporaneous values (as in Models 1) improves the forecasting accuracy of industrial land. For the years 2006 and 2008, TAE and RD have smaller values regarding Models A2, B2 and C2 (Table 10) compared to their counterparts provided for Models 1 (Table 9). For the same years, RD is considerably smaller in Models 2 ranging from -0.07 to 0.026 than those in Models 1which range from -4.31 to 2.21.

**Fig 3:**
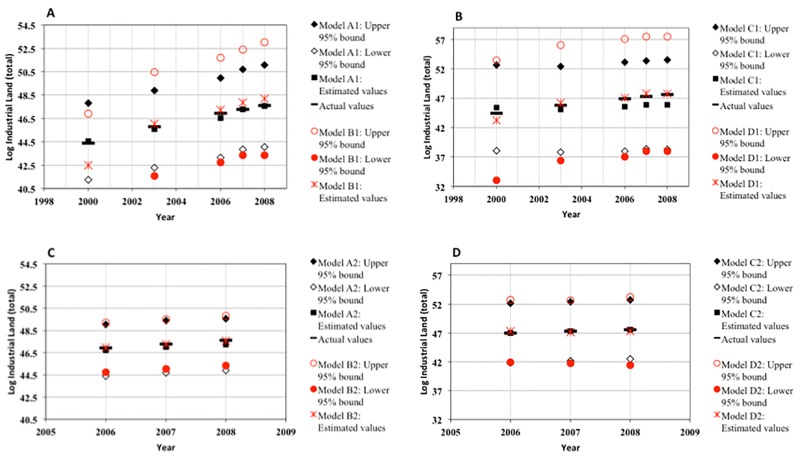
Industrial land use 2000–2008, with 95% confidence limits (sum of all Netherlands regions). A: Models A1 and B1. B: Models C1 and D1. C: Models A2 and B2, 2006–2008. D: Models C2 and D2, 2006–2008.

The models can also be evaluated based on the prediction interval forecasts. Basically, a prediction interval forecast is an interval of values that is calculated according to a specified level of confidence where the estimated true value lies within the confidence intervals. The results for Models 1 are depicted in Fig 3A and 3B whereas Fig 3C and 3D present the results for Models 2. For the NUTS3 region totals, Fig 3 shows the actual amount of industrial land, the estimates of the true values of industrial land, and lower and upper limits of the 95% confidence interval. For example, in Fig 3A the prediction interval for 2003 for Model B1 implies that with a confidence level of 95% the estimated true value of industrial land will fall between 41.5 and 50.5. From Fig 3C and 3D, Models C2 and D2 have wider confidence intervals than those observed for Models A2 and B2. This implies that Models C2 and D2 are more sensitive to uncertainties regarding the true value of industrial land compared to the latter models.

**Fig 4:**
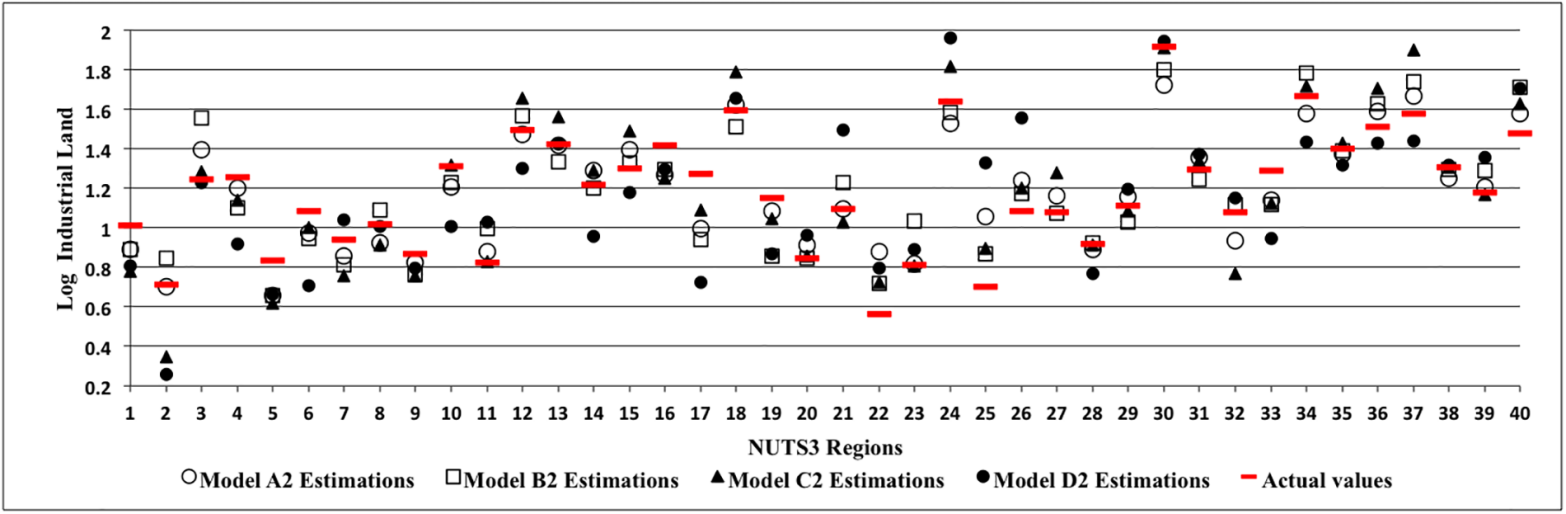
Comparison of actual and predicted (from Models 2) industrial land use in 2008 per region.

The predictions for each NUTS3 region generated by the second group of models are plotted in Fig 4 in comparison to the actual industrial land-use data. From Fig 4, there is no single model which can estimate the true value of industrial land with high levels of accuracy for all the NUTS3 regions. For regions 4, 5, 6, 9, 16, 17, 19 and 33 (South-West, South-East and North Friesland, South West Drenthe, Arnhem-Nijmegen, Flevoland, Kop North Holland, Overig Zeeland) all the models underestimate industrial land while for regions 11, 22, 23, 25, 26, 27 and 40 (South West Overijssel, Haarlem, Zaanstreek, Het Gooi-Vechtstreek, Leiden-Bollenstreek, The Hague, South Limburg) the models overestimate the true value (see Fig 1 and Table 1 for the details of regions). Regarding all NUTS3 regions, Models A2 and B2 generated closer estimates of industrial land to the actual land values compared to Models C2 and D2 predictions.

**Fig 5:**
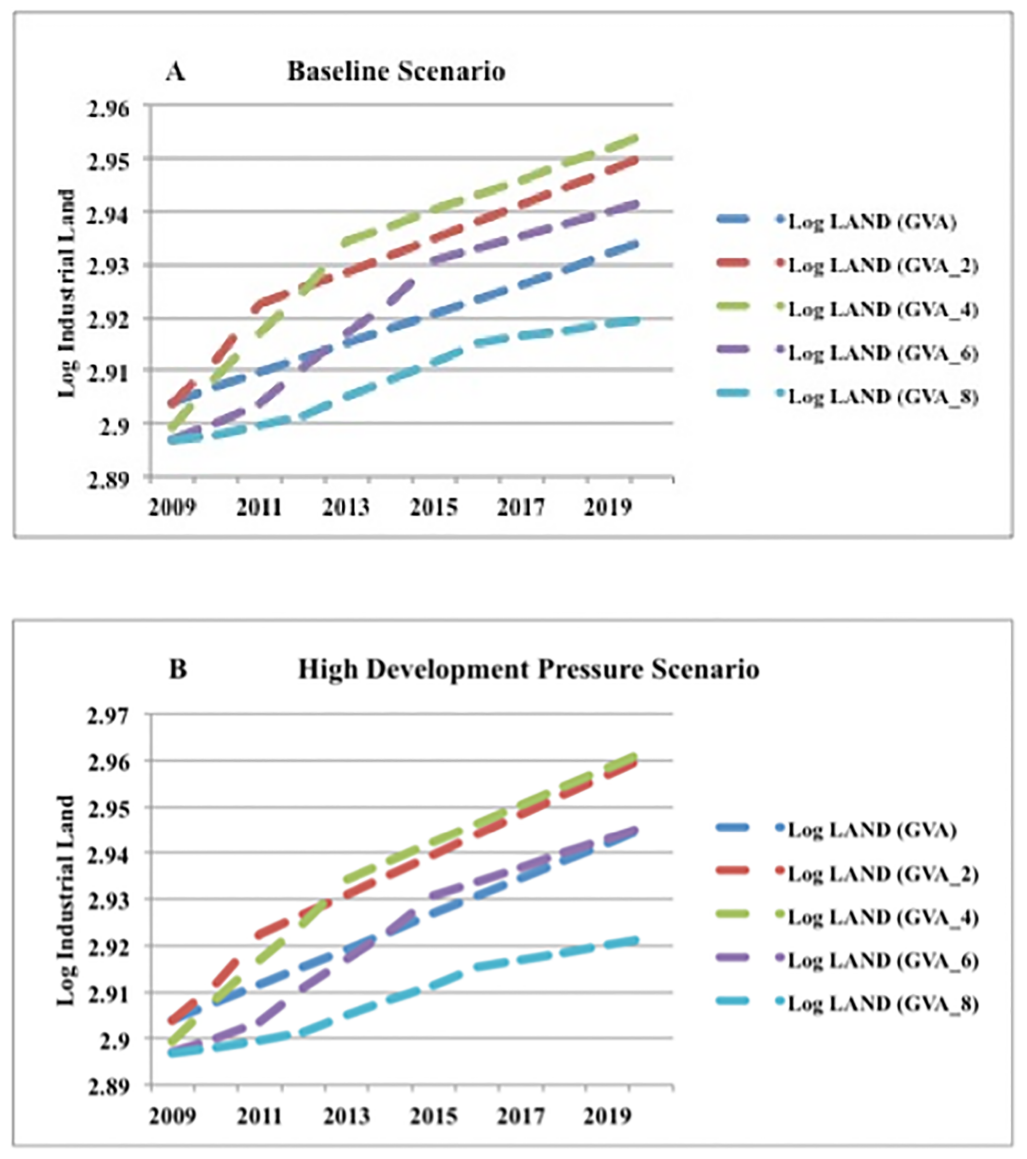
Scenarios of future demand for industrial land in the Netherlands.

Finally, we forecasted future land-use demand for the Netherlands based on two alternative scenarios to illustrate the differences of the use of different lagged values of economic parameters on future demand for industrial land. The scenarios consist of a baseline scenario where economic growth trends are derived from OECD baseline scenario [102] linked with the study ‘The Netherlands in a Sustainable World’, by the Netherlands Environmental Assessment Agency; and an alternative High Development Pressure scenario, which is based on the Global Economy scenario in the study ‘Welfare, Prosperity and Quality of the Living Environment’ [103]. The latter scenario assumes large numbers of economic migrants from new member states into the Netherlands. The details of the scenarios can be seen in ‘The Netherlands in the Future Second Sustainability Outlook Report’ [103]. From the Report, the forecasted annual growth rate of GDP is 1.9 percent for the baseline scenario and 2.6 percent for the High Development Pressure scenario between 2002 and 2040. These growth rates were applied to obtain the future values of GVA for the Netherlands for the 2009–2020 period, and utilised to forecast industrial land demand from the regressions that we estimated to identify the impact of individual lags of the GVA variable on industrial land use (Table 6). The forecasted industrial land demand from this analysis is presented in Fig 5. From Fig 5(b), High Development Pressure scenario results in higher industrial land particularly when contemporaneous value, and second and fourth lags of GVA are used in the land demand forecasting. Both Fig 5(a) and 5(b) demonstrate that industrial land demand is the lowest when eight-period lag of GVA is utilised in the analysis. It is also confirmed in Table 6 that the estimated coefficient of eight-period lag of GVA is the smallest compared to others. The trends observed in Fig 5 indicate uncertainties in the projected industrial land use depending on the use of different lagged values of GVA in industrial land forecasting work. This implies that land-forecasting studies focusing on a single economic parameter for the projection of future land demand should consider the uncertainties stemming from the use of different lagged parameters in the forecasting analysis.

In future projection of industrial land demand, we have considered socio-economic forces as they were embedded in the alternative scenarios [103] that were developed to forecast the values of economic parameters (e.g. GDP). However, individual characteristics of industrial firms in the post-2016 period, which are different than existing firms, are not captured in the current analysis. During recent decades, advanced technologies accompanied by advanced knowledge and innovation systems have been emerging with an increasing pace. An example for an advanced production systems is ‘additive manufacturing (or 3-D printing)’ which is characterised by a process where new locations of economic activity and innovation emerge following an unprecedented change in technology [104]. This process is seen to be sensitive to provision of services, firm scale, clustering and networks [105]. Gress and Kalafsky [106] noted that for an effective production, additive manufacturing may be the catalyst for the movement of such manufacturing closer to the research sources. The existing advanced knowledge synergies and networking may reinforce the existing clusters, and together with new innovations, existing clusters could be transformed to house the activities of advanced manufacturing [107]. ‘This is happening in geographically centralised manufacturing centres including Belgium, Germany and the Netherlands’ [106]. Innovation and advanced manufacturing may take place in other locations as well through the construction of new knowledge synergies, innovation and networking.

EC ([110]: 6) identifies ‘environmental’ and ‘organisational’ factors that lead high-tech firms’ location decisions with respect to production, R&D, and innovation. Environmental factors comprise: a) location characteristics (input cost factors, social factors, scientific and technological strengths, political stability); b) industry, sector, value chain characteristics (the value of being near an ecosystem with suppliers or other firms in the same sector); c) market considerations (proximity with important markets and key customers). Likewise, organisational factors consist of: a) strategic considerations (firms’ strategy with respect to market entry and growth, product/market combinations, production organisation, distribution and services); b) technology and innovativeness (intensive collaboration between production, R&D and innovation and a more pro-active strategy towards knowledge and technology sourcing); c) product and production complexity (has a strong influence on the need to co-locate production with development and research activities).

Concerning the representation of location decisions of firms using advanced manufacturing techniques in the analysis of industrial land development, it is useful to analyse the existing clusters of high-tech firms, research and innovation centres, their knowledge synergies and networking among them. The size and geographical location of these clusters and highly-skilled employment working in these high-tech industries are crucial parameters that could be included in the analysis to identify the location and land-use requirements of high-tech industries, research and innovation centres. Future projections on demand for advanced manufacturing outputs, geographies of consumption, required services to support advanced manufacturing industries, geographies of labour, innovation and R&D facilities are required to be incorporated in the projection of industrial land regarding the land requirements of high-tech production activities. We leave this as a future research topic depending on the accessibility of required data on the geographies of advanced manufacturing and innovation systems to be included in the analysis of industrial land use in the Netherlands.

## Discussion and conclusions

The findings of this study are policy relevant, as many forecasting models for real property demand analysis are using socio-economic indicators to predict industrial land development. The existence of time lag effects concerning particularly the socio-economic parameters is important to be considered in the models that focus on prediction of demand for industrial property. It is of particular significance for the land-use models where a reliable estimation of future land demand is required to be integrated into spatial land-use models. It is also important for policy makers and planners to formulate timely and forward-looking strategies to distribute and allocate scarce land resources efficiently. While previous research on real estate demand forecasting focused on cross-section data of the socio-economic parameters- that are limited to reflect cyclical fluctuations, it is important to develop reliable forecasting models which can provide the impact of economic fluctuations on land-use development. The results of this study should provide a basis for researchers, planners and policy makers to explore the applicability of our dynamic panel regression technique in modelling industrial land demand during the periods of economic boom or recession. The use of time lag effects by the model could serve as a useful source for construction and industry stakeholders to implement appropriate strategies during periods of cyclical fluctuations.

There are two key findings of the study: First, our models highlight the importance of economic output, national income, industrial property price and employment in influencing industrial land developments. In this, it is shown that fluctuations in the economic fundamentals are closely interrelated to similar fluctuations in the development of industrial land. This is in line with the findings of the economic literature outlined previously. Second, our findings suggest that industrial developments are mainly explained by the existence of time lags between the changes in industrial property price, industrial GVA (or GVA to GDP ratio) and employment, and the changes in industrial land developments. Considering time lags between demand and supply which can be related to inefficiencies of the land markets in the Netherlands, fluctuations in demand for industrial property are not immediately reflected in industrial developments.

Our dynamic panel regression models highlight the varying contribution of economic fundamentals to the industrial land development at different times. The models also successfully capture the regional and market-based heterogeneity at different times throughout the use of NUTS3 regions as the spatial unit of the analysis and through the utilisation of instrumental variables in the dynamic panel regressions that can represent socio-economic and location specific characteristics of the regions. Therefore, our modelling approach provides an improvement over the time-series models discussed in section 2.2. However, there are still issues to be covered in our modelling approach such as the inclusion of agglomeration, spillover effects and networking representing the knowledge synergies of high-tech industrial developments on land use. Future research can make a contribution to the model by focusing on such impacts to explain regional variations in industrial land developments.

Concerning industrial property price indicator, in contrast to the lagged values, the current value of property price is the key driver of industrial developments as it has larger coefficients compared to those with two to six-period lags. This implies that property price can be considered as a short-term indicator in the Dutch industrial property market in such a way that it gives signals to property developers whether to invest in a new industrial development scheme requiring expansion of industrial land. A further implication of the findings suggests that industrial property prices can be used to forecast short to medium-term demand for industrial land. However, the indicator is insufficiently robust to forecast the long-term future demand of industrial land use.

Although the first group of models find significant coefficients for the current values of output and income indicators, our results suggest caution with the consideration of industrial GVA and GVA/GDP in current values (as in Table 5) to forecast the demand for industrial space. A further issue is that contemporaneous values of these predictors in the second group models can explain only a small share of the variation in industrial land demand considering the existence of lagged effects of the predictors in industrial developments. Therefore, consideration of lagged values as well as the current values of economic indicators in the industrial land forecasting work will improve the robustness of the analysis. Furthermore, our models suggest that output and income indicators can appropriately explain short to long-term (up to eight years) relationship between the lagged values of subject indicators and industrial land use. This is crucial to be considered in the studies which focus on economic indicators (i.e. GVA, GDP) to forecast future land demand. The examples include Reginster and Rounsevell [41], Rounsevell et al. [14], Hoymann [13], and Batista e Silva et al. [10] among others.

A significant issue to be considered in the forecasting models is the influence of economic shocks on the development structure of industrial land. The post-2006 crisis has changed the structural relationship between economic parameters and land use. Therefore, the possibility of an economic shock in relation to the persistence of its effects to the time series plays a role in the demand-supply relationship of the industrial property. A further issue to be considered is the unprecedented reduction of population and employment in some regions in Netherlands which will come in the succeeding decades following a long period of uninterrupted growth [108]. This may lead to structural changes in labour and real property markets considering that business sectors may adopt their demand for industrial property and their location choice to the new economic conditions [18]. This would result in unprecedented effects on the long-term demand for industrial real estate. It is therefore important to update the models at various times in such a way that unforeseeable changes are fully reflected and the model forecasts are generated with high accuracy. For this reason, land forecasting applications should consider the influences of shocks in the economy and structural changes in labour and real property markets through the consideration of full time series data particularly for the post-2006 period.

The forecasting models have been increasingly used by national and local planning authorities to predict and allocate land uses on various sites in different regions. In particular, this is of great significance for the Netherlands due to the physical limitations of land development in the country, and due to the existence of strict institutional and regulatory structures influencing industrial land markets. We should note that the research presented here is not confined to the Netherlands and should be extended to cover other regions and countries in terms of further improvements in the methodology and applications with regard to industrial land analysis. However, it should be also noted that the application of current analysis requires long-term time-series spatial data regarding industrial land-use. Though long-term socio-economic data at the regional level is widely available in most countries, there are data accessibility issues for the time-series spatial data. This may limit application of the current study for other countries at the regional or local levels.

Though our model is able to incorporate spatial heterogeneities observed across the regions in the study area, the model cannot capture the spatial dependencies observed at neighbouring locations. As an alternative methodology for introducing spatial lag effects -that is based on the notion that the value of a variable at a given location is related to the values of the same variables measured at nearby locations, we refer to spatial error modelling approach, also known as spatial autoregressive model. This methodology can be used as an alternative approach in the future work for the prediction of demand for industrial land. However, it is important to mention that the model is unable to incorporate time lag effects of the dependant and explanatory variables. Therefore, a methodological development is necessary regarding the spatial error models to account for the impacts of time lags of the dependant and selected independent variables. This will be a challenging work as sophisticated techniques are required for methodological improvement.

## Appendix

Given the assumption of serially uncorrelated residuals, values of ‘y’ lagged two periods or more can be used as instruments in the first differenced equation [101]. This implies that the moment conditions use the properties of the instruments y_i,t-j_, j≥2 to be uncorrelated with the future errors ε_i,t_ and implying the following moment conditions:

$$E\left(y_{i,t-j},\Delta \varepsilon_{i,t}\right)=0;t=3,\ldots ,T;j\geq 2$$ (A1)

All explanatory variables which are strictly exogenous can also be used as instruments. In this, it is assumed that x is weakly exogenous rather than strictly exogenous, which implies the following condition (see Greene [101] for the details):

$$E\left(\Delta x_{i,t-j}\Delta \varepsilon_{it}\right)=0;t=3,\ldots ,T;j\geq 2$$ (A2)

This leads to the matrix Z_i_ i.e. the matrix of instrumental variables:

$$Z_{i}=\left[\begin{array}{cccc} y_{i1},x_{i1},x_{i2} & 0 & \ldots & 0\\ 0 & y_{i1},y_{i2},x_{i1},x_{i2},x_{i3} & \ldots & 0\\ \vdots & \vdots & \ddots & \vdots \\ 0 & 0 & \ldots & y_{i1},y_{i2},\ldots ,y_{i,T-2},x_{i1},x_{i2},\ldots ,x_{i,T-1} \end{array}\right]$$ (A3)

The GMM approach discussed so far utilises the moment conditions set by the Eqs (A1) and (A2) given the first-differenced Eq (5). The consistency of the GMM estimators relies on the correct specification of the model and the moment conditions, which in turn, are influenced by the first-differenced disturbances being serially uncorrelated and the explanatory variables being exogenous to the system of equations.

## Supporting information

S1 File Panel data for the analysis of Netherlands. (XLS)
